# Sex Hormones and Aging Modulate Interferon Lambda 1 Production and Signaling by Human Uterine Epithelial Cells and Fibroblasts

**DOI:** 10.3389/fimmu.2021.718380

**Published:** 2021-09-24

**Authors:** Mickey V. Patel, Daniel C. Hopkins, Fiona D. Barr, Charles R. Wira

**Affiliations:** Department of Microbiology and Immunology, Geisel School of Medicine at Dartmouth, Lebanon, NH, United States

**Keywords:** interferon lambda (IFN-λ), estradiol, progesterone, uterine epithelial cell, uterine stromal cell, interferon-stimulated gene (ISG)

## Abstract

Estradiol (E_2_) and progesterone (P) have potent effects on immune function in the human uterine endometrium which is essential for creating an environment conducive for successful reproduction. Type III/lambda (λ) interferons (IFN) are implicated in immune defense of the placenta against viral pathogens, which occurs against the backdrop of high E_2_ and P levels. However, the effect of E_2_ and P in modulating the expression and function of IFNλ1 in the non-pregnant human uterine endometrium is unknown. We generated purified *in vitro* cultures of human uterine epithelial cells and stromal fibroblast cells recovered from hysterectomy specimens. Poly (I:C), a viral dsRNA mimic, potently increased secretion of IFNλ1 by both epithelial cells and fibroblasts. The secretion of IFNλ1 by epithelial cells significantly increased with increasing age following poly (I:C) stimulation. Stimulation of either cell type with E_2_ (5x10^-8^M) or P (1x10^-7^M) had no effect on expression or secretion of IFNλ1 either alone or in the presence of poly (I:C). E_2_ suppressed the IFNλ1-induced upregulation of the antiviral IFN-stimulated genes (ISGs) MxA, OAS2 and ISG15 in epithelial cells, but not fibroblasts. Estrogen receptor alpha (ERα) blockade using Raloxifene indicated that E_2_ mediated its inhibitory effects on ISG expression *via* ERα. In contrast to E_2_, P potentiated the upregulation of ISG15 in response to IFNλ1 but had no effect on MxA and OAS2 in epithelial cells. Our results demonstrate that the effects of E_2_ and P on IFNλ1-induced ISGs are cell-type specific. E_2_-mediated suppression, and selective P-mediated stimulation, of IFNλ1-induced ISG expression in uterine epithelial cells suggest that the effects of IFNλ1 varies with menstrual cycle stage, pregnancy, and menopausal status. The suppressive effect of E_2_ could be a potential mechanism by which ascending pathogens from the lower reproductive tract can infect the pregnant and non-pregnant endometrium.

## Introduction

Unique among mucosal sites, the immune system in the uterine endometrium has evolved to protect against incoming pathogens while creating an environment essential for successful reproduction. Key to this are the sex hormones estradiol (E_2_) and progesterone (P) whose concentrations change across the menstrual cycle, during pregnancy, and following menopause. The changing levels of hormones regulate multiple aspects of the innate and adaptive immune systems in the female reproductive tract (FRT), and particularly in the uterine endometrium, to allow for successful fertilization, implantation, and survival of a semi-allogeneic fetus (1). However, changes in immune function due to changing hormone levels are linked to altered susceptibility to sexually transmitted infections (STIs) (1), which can negatively impact reproduction.

Interferons (IFNs) encompass three different families of cytokines (I, II, and III) and are essential for preventing infections. The Type III IFNs are a family of antiviral cytokines consisting of IFNλ1, λ2, λ3 and λ4 (2–4) that are produced in response to pathogens. Previous studies have shown that stimulation of pattern recognition receptors (PRR) such as Toll-like receptor 3 (TLR3) and TLR9 increase the secretion of IFNλ1-3 *in vitro* (5–7). In turn, Type III IFNs exert their effects in an autocrine and paracrine fashion *via* a heterodimeric receptor complex consisting of IL28Rα (IFNLR1) and IL10Rβ (IL10R2) (2, 3). Similar to the Type I IFNs such as IFNβ, Type III IFN receptor activation initiates JAK/STAT signaling that upregulates expression of downstream IFN stimulated genes (ISG) such as Myxovirus A (MxA), Oligoadenylate Synthetase (OAS) 1-3, and ISG15. These ISGs can inhibit different stages of the viral lifecycle, thus creating an intracellular antiviral state hostile to pathogen survival. While previous studies have shown that sex hormones can modulate the secretion of, and sensitivity to, Type I IFNs in dendritic cells (8, 9), their effect on Type III IFNs is relatively unknown.

Type III IFNs function primarily at mucosal surfaces, in contrast to the Type I IFNs that induce antiviral responses throughout the body (10). For example, murine intestinal epithelial cells lacking functional Type III IFN receptors are more susceptible to rotavirus infection than wild-type mice (11). Type III IFNs increased the resistance of respiratory epithelial cells against influenza A virus and severe acute respiratory syndrome coronavirus, while IFNλ1 reduced infection of nasal epithelial cells by respiratory syncytial virus (12, 13). However, our understanding of Type III IFNs in the human FRT mucosa is sparse, with research focusing on the lower FRT (vagina and ectocervix). In the murine vagina, CD11c+ dendritic cells reduced Herpes Simplex Virus (HSV) 2 infection *via* the secretion of IFNλ1 (14), while induction of IFNλ1 in the End1/E6E7 human cervical epithelial cell line also inhibited HSV-2 (15). Differences in IFNλ1, IL28Rα, and IL10Rβ expression in human cervical epithelial cells is thought to be associated with low- *versus* high-risk Human Papilloma Virus (HPV) progression (16). Type III IFNs are also essential for the immune defense of the placenta against viral pathogens such as Zika Virus (ZIKV) (17, 18). Recognizing that the uterine endometrium is an anatomical compartment with a unique immunological environment that is distinct from the lower FRT (1, 19, 20), there exists a particular need to understand the contribution of Type III IFNs to innate immune protection at this unique site.

In this study we used IFNλ1 as model to study the effects of sex hormones and age on the secretion of, and sensitivity to, Type III IFNs by uterine epithelial cells and fibroblasts, the major non-hematopoietic cell types at the uterine mucosal surface. Both uterine cell types secrete IFNλ1 in response to the dsRNA viral agonist, poly (I:C), independent of E_2_ and P. However, E_2_ suppressed IFNλ1-induced upregulation of the antiviral genes OAS2 and ISG15 in epithelial cells *via* estrogen receptor alpha (ERα) signaling. P potentiated the upregulation of ISG15 by epithelial cells following treatment with IFNλ1 but had no effect on MxA and OAS2. In contrast, neither E_2_ nor P had any effect on IFNλ1-induced gene expression in stromal fibroblasts. These results demonstrate that the effects IFNλ1 vary by cell type and hormone exposure in the human uterine endometrium while also implicating E_2_ and P as regulators of IFNλ1 signaling.

## Materials and Methods

### Source of Uterine Tissue

Human uterine tissue was obtained from women (28-81 years old) undergoing hysterectomy surgery at Dartmouth-Hitchcock Medical Center (Lebanon, NH). Reasons for surgery were menorrhagia, adenomyosis, adnexal mass, fibroids and prolapse. All tissues used in this study were distal to the sites of pathology and were determined to be unaffected with disease upon inspection by a pathologist. Tissues were excluded under conditions of cancer. All investigations involving human subjects were conducted according to the principles expressed in the Declaration of Helsinki and carried out with the approval from the Committee for the Protection of Human Subjects (CPHS), Dartmouth Hitchcock Medical Center, and with written informed consent obtained from the patients before surgery.

### Isolation of Uterine Epithelial Cells and Uterine Fibroblasts

Tissues were minced under sterile conditions into 1 to 2mm fragments and subjected to enzymatic digestion using an enzyme mixture containing 0.05% collagenase type IV (Sigma-Aldrich, St. Louis, MO) and 0.01% DNAse (Worthington Biochemical, Lakewood, NJ) in 1x HBSS (Invitrogen). After enzymatic digestion for 1 hr at 37°C, cells were dispersed through a 250-µm mesh screen, washed, and resuspended in Hank’s Balanced Salt Solution (ThermoScientific, Logan, UT).

Epithelial cell sheets were separated from stromal fibroblasts by filtration through a 20-µm nylon mesh filter (Small Parts, Miami Lakes, FL). Epithelial sheets were retained on the 20-µm filter, while the stromal fraction containing the fibroblasts passed through and were collected as part of the filtrate. Epithelial sheets were recovered by rinsing and backwashing the filter with Complete medium, centrifuged (500 x *g*, 10 min), and analyzed for cell number and viability.

### Uterine Epithelial Cell and Uterine Fibroblast Cell Culture

To establish an *in vitro* cell culture system of polarized human uterine epithelial cells, uterine epithelial cells were cultured in Falcon cell culture inserts coated with Human Extracellular Matrix (Becton Dickinson, Franklin Lakes, NJ) in 24-well culture plates (Fisher Scientific, Pittsburgh, PA). Apical and basolateral compartments had 300 and 500 µl of complete medium respectively. Complete medium consisted of DMEM/F12 supplemented with 20 mM HEPES (Invitrogen), 2 mM L-glutamine (Invitrogen), 50 mg/ml primocin (Invivogen) and 10% heat-inactivated defined Fetal Bovine Serum (FBS) (ThermoScientific). Incubation media was changed every 2 days.

To establish a purified population of uterine stromal fibroblasts, the stromal filtrate was centrifuged (500 x *g*, 10 min) and the pellet resuspended in complete media. Cells were placed in culture in a 75 cm^2^ cell culture flask (Fisher Scientific) in complete medium until they reached confluence. The medium was changed every 2 days. After reaching confluence, cells were trypsinized and 1x10^6^ cells added to a fresh 75 cm^2^ flask. This was repeated at least twice, after which cells were recovered and plated (1x10^5^ cells/well) in 24-well cell culture dishes (Fisher) in 500 μl of complete medium with charcoal dextran-stripped FBS for at least 48 hrs prior to treatment. Sequential passaging allows the fibroblasts to outcompete any other cell types present in the original stromal filtrate, leaving a population of fibroblasts that are positive for the stromal fibroblast markers vimentin and CD90, and negative for the hematopoietic cell marker CD45 as previously described (21, 22).

### ECC-1 Cell Culture

The ECC-1 cell line (originally established by Dr. Pondichery Satyaswaroop and kindly provided by George Olt, Penn State College of Medicine, Milton S Hershey Medical Center, PA) is a human uterine epithelial cell line, characteristic of the luminal uterine epithelium, that is responsive to sex hormones (23) and is used by us to study innate immune responses of FRT epithelial cells. To establish a culture system of polarized human ECC-1 cells with both apical and basolateral compartments, ECC-1 cells were cultured in uncoated Falcon cell culture inserts in 24-well culture dishes (Fisher). Apical and basolateral compartments had 300 and 500 μl of complete medium respectively as described above for primary cells.

### Measurement of Transepithelial Resistance

As an indicator of tight junction formation and polarization of uterine epithelial cell monolayers, transepithelial resistance was assessed every 48 hrs using an EVOM electrode and Voltohmmeter (World Precision Instruments Inc., Sarasota, FL) as described previously (24). Matrigel-coated inserts without any cells were used as controls and had transepithelial resistance values of 200-240 ohms.

### Poly (I:C), Interferon λ1, Estradiol, and Progesterone Stimulation

Cells were stimulated with HMW-poly (I:C) (Invitrogen) at 0.25-25 µg/ml in complete media with 10% defined FBS, except as noted below, for up to 24 hrs. Recombinant human IFNλ1 (PBL Assay Science, Piscataway, NJ) was used at 1-1000 ng/ml in complete media with 10% charcoal dextran-stripped FBS (Gemini, West Sacramento, CA) for up to 24 hrs. For polarized primary epithelial and ECC-1 cells, poly (I:C) was added to the apical compartment only, while IFNλ1 was added to both the apical and basolateral compartments.

Type I IFN receptor blockade experiments used a mouse monoclonal anti-human interferon receptor 2 (IFNAR2) blocking antibody (R&D) or a matched IgG2A isotype control at a final concentration of 10μg/ml for 1 hr and then stimulated with poly (I:C) for 24 hrs (25). Type III IFN receptor blockade experiments used a mouse monoclonal anti-human αIL10Rβ blocking antibody (R&D). In all experiments, blocking antibody was maintained in the culture media (complete media with 10% charcoal dextran-stripped FBS) throughout the experiment.

For hormone experiments, E_2_, P, or the estrogen receptor (ER) antagonist Raloxifene (Rx) (Calbiochem, Gibbstown, NJ) were dissolved in 100% ethanol for an initial concentration of 1x10^-3^M, evaporated to dryness and resuspended in complete media containing charcoal dextran-stripped FBS to a concentration of 1x10^-5^M, as previously described (21, 25, 26). Further dilutions were made to achieve final working concentrations of E_2_ ranging from 5x10^-8^M to 5x10^-10^M. P was used at 1x10^-7^M. As a control, an equivalent amount of 100% ethanol without dissolved hormone was initially evaporated. For polarized uterine epithelial cells and ECC-1 cells, E_2_ or P was added to both the apical and basolateral compartments. For ER blockade experiments, cells were pretreated with Raloxifene, a selective estrogen response modulator that antagonizes estrogen effects in uterine cells (27) and has been used by us in other studies (28), at 5x10^-6^M (100-fold excess) for at least 1 hr prior to the introduction of E_2_. Raloxifene was maintained in the cell culture media throughout the experiment.

For hormone experiments, and receptor-blockade experiments, cells were transferred from complete medium with 10% defined FBS to complete medium with 10% charcoal dextran-stripped FBS for at least 48 hrs prior to hormone treatment to remove the influence of any steroid hormones present in defined FBS that could otherwise confound the effects of exogenous E_2_ or P.

### TaqMan Real-Time RT-PCR

Total mRNA was isolated and purified using a RNeasy mini kit (Qiagen, Valencia, CA) with on-column DNase digestion using the RNase-Free DNase set (Qiagen) according to the manufacturer’s recommendations. 400ng of total RNA was reverse-transcribed using the iScript cDNA synthesis kit (Bio-Rad) according to the manufacturer’s recommendations. Relative mRNA expression levels of IFNλ1 (Hs00601677_g1), MxA (Hs00895608_m1), OAS2 (Hs00942643_m1), ISG15 (Hs01921425_m1), IL28Rα (Hs00417120_m1), and IL10Rβ (Hs00175123_m1) were measured using the 5’ fluorogenic nuclease assay in real-time quantitative PCR using TaqMan chemistry on the ABI 7300 Prism real-time PCR instrument (Applied Biosystems, Carlsbad, CA). PCR was conducted using the following cycle parameters: 50°C, 2 mins, 1 cycle; 95°C, 10 mins, 1 cycle; 95°C, 15 s, 40 cycles; 60°C, 1 min, 1 cycle. Analysis was conducted using the sequence detection software supplied with the ABI 7300. In several patients, the expression of IFNλ1 was undetectable in untreated samples. In these cases, the expression of IFNλ1 was assigned a Ct value of 40, which is the lowest possible value in our system, for the purposes of calculating the fold-increase in IFNλ1 mRNA expression following treatment with poly (I:C).

Data are presented either as relative expression normalized to β-Actin, or as fold-change in mRNA expression. When presented as relative expression, the expression of the gene of interest is normalized to the expression of housekeeping gene β-Actin. Data are also presented as fold change in mRNA expression, where fold change refers to the change in expression of a gene of interest between the control and treatment group(s) which are both normalized to β-Actin expression and calculated using the formula $2_{}^{-\Delta \Delta \text{C}}$ _t_. When expressing our data as fold change, the control or untreated group is set to 1. For the purposes of clarity, in some cases the control group is absent from the fold change figures. However, this is noted in both y-axis legends and the figure legends.

### Cytokine and Antimicrobial Measurement

Following treatment, conditioned media were recovered from epithelial cells (apical and basolateral) and fibroblasts and centrifuged at 10,000 x g in a microfuge. Supernatants were aliquoted and stored at -80°C. ELISA was used to determine the secretion of IFNλ1, CCL4, elafin, CCL20, RANTES (all R&D Systems, Minneapolis, MN), and HBD2 (Peprotech, Cranbury, NJ) by uterine epithelial cells and fibroblasts according to the manufacturer’s instructions.

### Statistical Analysis

Data analysis was performed using the GraphPad Prism 5.0 (GraphPad Software, La Jolla, CA). A two-sided P value <0.05 was considered statistically significant. Comparison of treatment groups *vs*. control group was performed applying Mann-Whitney U test for non-matched samples or Wilcoxon matched-pairs signed rank test for matched samples. Comparison of three or more groups was performed applying Kruskal-Wallis test for non-matched samples or Friedman test for matched samples, followed by Dunns-post test for multiple comparison correction. Correlations of age with protein secretion or mRNA expression were performed using the nonparametric Spearman correlation with a two-tailed p-value. Best-fit trend lines were generated using a simple linear regression.

## Results

### Poly (I:C) Induces IFNλ1 Expression and Secretion by Epithelial Cells

**Figure 1:**
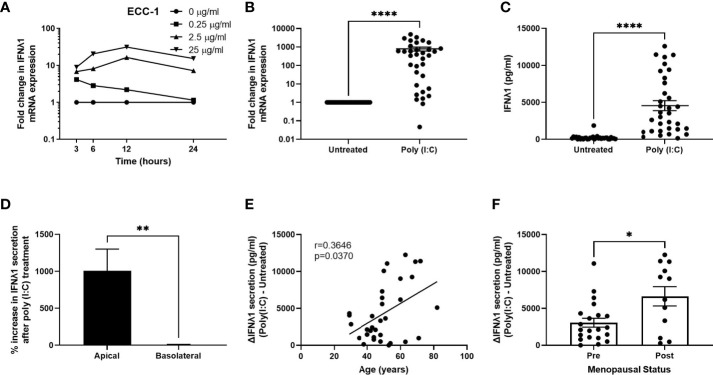
Poly (I:C) induces IFNλ1 expression and secretion by uterine epithelial cells. **(A)** ECC-1 uterine epithelial cells were stimulated with poly (I:C) for 3-24 hrs and IFNλ1 expression measured by RT-PCR. **(B, C)** Primary uterine epithelial cells were stimulated with poly (I:C) (25μg/ml) for 24 hrs prior to analysis of mRNA expression by real-time RT-PCR **(B)** and protein secretion **(C)** by ELISA. Each circle represents an individual patient (*n = 34*). **(D)** Percent increase in IFNλ1 secretion in the apical and basolateral compartments of transwell inserts following treatment of uterine epithelial cells with poly (I:C) (25μg/ml) for 24hrs *versus* untreated wells (*n = 34*). **(E, F)** Values for IFNλ1 secretion from panel E by poly (IC)-treated epithelial cells were subtracted from untreated wells to determine the difference in IFNλ1 secretion between matched poly (I:C)-treated and untreated wells (ΔIFNλ1 pg/ml) which was then plotted against patient age **(E)** (*n = 34*) or menopausal status **(F)** (*n = 34*). Each circle represents an individual patient. Data is shown as mean +/- SEM. Wilcoxon matched-pairs signed rank test **(B–D)**. Non-parametric Spearman correlation analysis **(E)**. Mann-Whitney non-parametric t-test **(F)**. *p < 0.05; **p < 0.01; ****p < 0.0001.

We have previously shown that stimulation of FRT epithelial cells and stromal fibroblasts with the synthetic dsRNA viral ligand poly (I:C) induces a potent innate immune response (21, 22, 25, 26, 29, 30). However, whether IFNλ1 is part of the innate immune response of uterine epithelial cells and fibroblasts in response to viral pathogens is unknown. As part of our initial studies, we used uterine ECC-1 epithelial cells. This uterine epithelial cell line, which we have used extensively, displays some of the innate immune characteristics of primary uterine epithelial cells, including polarization and recognition of poly (I:C) (25, 30). We treated polarized uterine ECC-1 epithelial cells with multiple doses of poly (I:C) (0.25, 2.5 & 25 μg/ml) for 3-24 hrs. Poly (I:C) dose-dependently increased the expression of IFNλ1 mRNA in ECC-1 cells with maximum levels reached between 12-24 hrs after stimulation with 25μg/ml (**Figure 1A**). Based upon these results, we treated primary uterine epithelial cells with poly (I:C) for 24 hrs. Following 24 hrs of poly (I:C) exposure, IFNλ1 mRNA expression was significantly upregulated by an average of 790-fold (**Figure 1B**) and IFNλ1 secretion in the apical compartment by approximately 4500 pg/ml (**Figure 1C**). Overall, constitutive apical IFNλ1 protein secretion by untreated samples was low with an average of 20.5 pg/ml. However, in many patient samples there was no detectable IFNλ1 secretion by untreated samples. In contrast to the significantly increased secretion of IFNλ1 in the apical compartment, there was no change in the levels of IFNλ1 in the basolateral compartment following poly (I:C) treatment (**Figure 1D**). There was a wide range in the increased expression and secretion of IFNλ1 between different patient samples following poly (I:C) exposure. For example, the fold change in epithelial IFNλ1 mRNA expression ranged between 0.04- to 4800-fold after 24 hrs of poly (I:C) treatment (**Figure 1B**) while the increased secretion of IFNλ1 ranged between 120-12300 pg/ml (**Figure 1C**).

### Poly (I:C)-Induced IFNλ1 Secretion by Epithelial Cells Increases With Age

We then calculated the difference in IFNλ1 secretion in the apical compartment between untreated cells and poly (I:C)-treated wells (ΔIFNλ1) and stratified the resulting values by patient age. As seen in **Figure 1E**, the poly (I:C)-induced secretion of IFNλ1 significantly increases with increasing age suggesting that the contribution of IFNλ1 to innate immune protection increases with age. We then stratified the results based on menopausal status. Consistent with our results in **Figure 1E**, we found that poly (I:C)-induced secretion of IFNλ1 was significantly higher by epithelial cells recovered from post-menopausal women compared to pre-menopausal women (**Figure 1F**).

### Poly (I:C) Induces IFNλ1 Expression and Secretion by Fibroblasts

**Figure 2:**
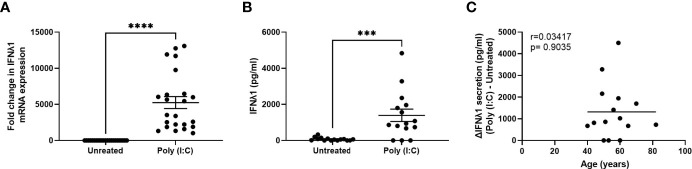
Poly (I:C) induces IFNλ1 expression and secretion by uterine stromal fibroblasts. Primary uterine fibroblasts were stimulated with poly (I:C) (25 μg/ml) for 24 hrs followed by analysis of **(A)** mRNA expression by real-time RT-PCR (*n = 23*) and **(B)** protein secretion by ELISA (*n = 15*). Each circle represents an individual patient. **(C)** Values for IFNλ1 secretion from panel B by poly (IC)-treated fibroblasts were subtracted from untreated wells to determine the difference in IFNλ1 secretion between matched poly (I:C)-treated and untreated wells (ΔIFNλ1 pg/ml) which was then plotted against patient age (*n = 15*). Data is shown as mean +/- SEM. Wilcoxon matched-pairs signed rank test **(A, B)**. Non-parametric Spearman correlation analysis **(C)**. ***p < 0.001; ****p < 0.0001.

We have previously shown that fibroblasts from throughout the FRT are capable of mounting a potent innate immune response following exposure to viral mimics such as poly (I:C) (21, 22, 26). To determine whether IFNλ1 was a part of this innate response, we stimulated primary uterine stromal fibroblasts with poly (I:C) for 24 hrs after which we measured IFNλ1 expression and secretion. There was a significant increase in IFNλ1 mRNA expression by an average of 5247-fold (**Figure 2A**) and secretion by 1390 pg/ml (**Figure 2B**) after 24 hrs. Similar to epithelial cells, constitutive IFNλ1 protein secretion by untreated samples was low, with no detectable IFNλ1 secretion in many samples. Furthermore, there was a wide range in both IFNλ1 mRNA expression (1014-13083-fold increase) and protein secretion (0-4832 pg/ml) following treatment with poly (I:C). However, in contrast to epithelial cells (**Figure 1E**), there was no effect of age on poly (I:C) induced secretion of IFNλ1 by fibroblasts (**Figure 2C**).

### Type I IFN Induces IFNλ1 Secretion by Uterine Epithelial Cells

**Figure 3:**
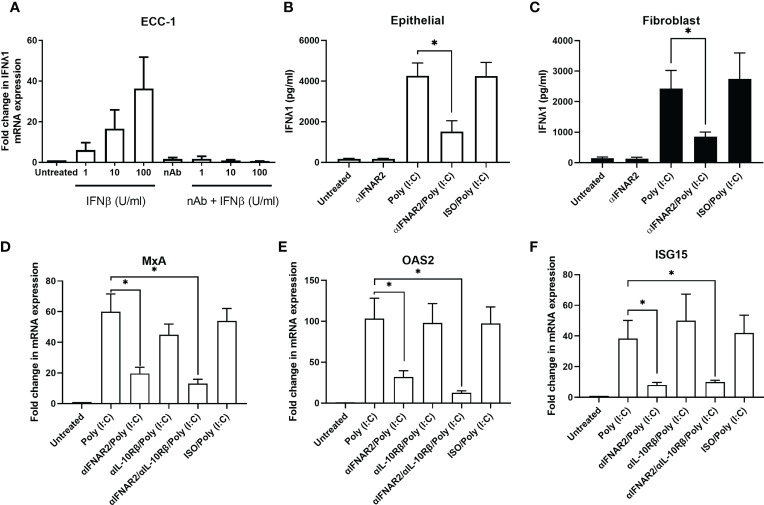
Blockade of Type I IFN signaling inhibits IFNλ1 secretions and signaling by uterine epithelial cells. **(A)** ECC-1 uterine epithelial cells were treated with recombinant human IFNβ for 24 hrs in the presence or absence of IFNβ neutralizing antibody after which IFNλ1 mRNA expression was determined (*n = 3*). **(B)** Uterine epithelial cells (*n = 6*) and **(C)** fibroblasts (*n = 6*) were pre-treated with a blocking antibody against IFNAR2 (αIFNAR2) or an isotype control (αISO) for 1 hr prior to the introduction of poly (I:C) (25 μg/ml) for 24 hrs, during which αIFNAR2 and αISO were maintained in the culture media. IFNλ1 secretion by epithelial cells **(B)** and fibroblasts **(C)** were determined by ELISA. Data is shown as mean IFNλ1 (pg/ml) +/- SEM. **(D)** Primary human uterine (*n = 6*) epithelial cells were pretreated with αIFNAR2, αIL-10Rβ, or a combination of both prior to treatment poly (I:C) for 24 hrs, after which mRNA expression of MxA **(D)**, OAS2 **(E)**, and ISG15 **(F)** was determined by RT-PCR. Data is shown as mean +/- SEM. Wilcoxon matched-pairs signed rank test **(B–F)**. *p < 0.05.

Previous studies have reported that Type I IFNs, such as IFNβ, can regulate Type III IFN expression (31). To investigate the potential role of IFNβ in regulating IFNλ1 in our system, ECC-1 cells were treated with recombinant human IFNβ for 24 hrs. IFNβ dose-dependently induced expression of IFNλ1, which was inhibited in the presence of an IFNβ neutralizing antibody (**Figure 3A**). Primary uterine epithelial cells or fibroblasts were then pre-treated with αIFNAR2, a blocking antibody directed against the IFNAR2 subunit of the heterodimeric Type I IFN receptor complex. When stimulated with poly (I:C), αIFNAR2 partially inhibited the upregulation of IFNλ1 secretion in both epithelial cells (**Figure 3B**) and fibroblasts (**Figure 3C**). This suggests that IFNλ1 expression in response to incoming poly (I:C) and viral stimuli is mediated partly *via* the Type I IFNs.

Building upon our past studies demonstrating that uterine epithelial cells upregulate ISGs in response to poly (I:C) (25), we investigated the contribution of IFNλ1 to this antiviral response by using a blocking antibody against IL10Rβ, one half of the heterodimeric IFNλ1 receptor complex. Primary uterine epithelial cells were pre-treated with αIL10Rb, αIFNAR2, or a combination of both prior to treatment with poly (I:C) for 24hrs after which MxA, OAS2, and ISG15 expression levels were measured. As seen in **Figures 3D–F**, blockade of Type I IFN signaling significantly decreased the expression of MxA, OAS2, and ISG15. However, blockade of Type III IFN signaling had no effect on MxA and OAS2 expression levels. When both Type I and Type III IFN signaling was blocked, expression of MxA, OAS2, and ISG15 was no different than Type I IFN alone. Together this suggests that Type I IFNs are the primary mediators of ISG expression by epithelial cells following exposure to poly (I:C).

### Estradiol and Progesterone Have No Effect on IFNλ1 Secretion by Epithelial Cells and Fibroblasts

**Figure 4:**
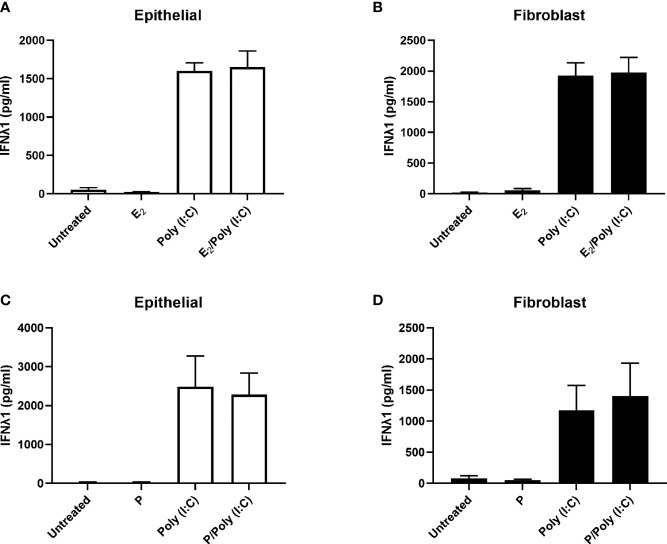
IFNλ1 secretion by uterine epithelial cells and uterine fibroblasts in response to poly (I:C) is independent of estradiol (E_2_) and progesterone (P). Primary human uterine epithelial cells **(A, C)** or uterine fibroblasts **(B, D)** were pretreated with E_2_ (5x10^-8^M) **(A, B)** or P (1x10^-7^M) **(C, D)** for 48 hrs after which media was replenished in the presence or absence of poly (I:C) (25 μg/ml) for a further 24 hrs. During this period E_2_ or P was maintained in the culture media. IFNλ1 secretion was determined by ELISA. Data is shown as mean +/- SEM. *n = 3* **(A, B)**. *n = 6* **(C, D)**.

Previous studies have shown that E_2_ can upregulate the secretion of secretory leukocyte protease inhibitor (SLPI) and inhibit IL-1β-mediated mRNA expression and secretion of human β-defensin-2 and CXCL8 by primary uterine epithelial cells (19). To determine if E_2_ regulates IFNλ1 secretion, we treated uterine epithelial cells, fibroblasts, and ECC-1 cells with E_2_ (5x10^-8^M – 5x10^-10^M) for up to 72 hrs. E_2_ did not alter the expression or secretion of IFNλ1 compared to untreated cells (data not shown). Pretreatment of uterine epithelial cells or stromal fibroblasts with E_2_ (5x10^-8^M) for 48 hrs followed by poly (I:C) stimulation also had no effect on IFNλ1 secretion by either cell type (**Figures 4A, B**). Similarly, we found that P (1x10^-7^M) either alone or in combination with poly (I:C) had no effect on the expression or secretion of IFNλ1 by epithelial cells and fibroblasts (**Figures 4C, D**). Together these studies indicate that sex hormones do not modulate the secretion of IFNλ1 by epithelial cells and fibroblasts in the uterine endometrium following exposure to viral pathogens.

### IFNλ1 Induces Gene Expression in Uterine Epithelial Cells and Fibroblasts

**Figure 5:**
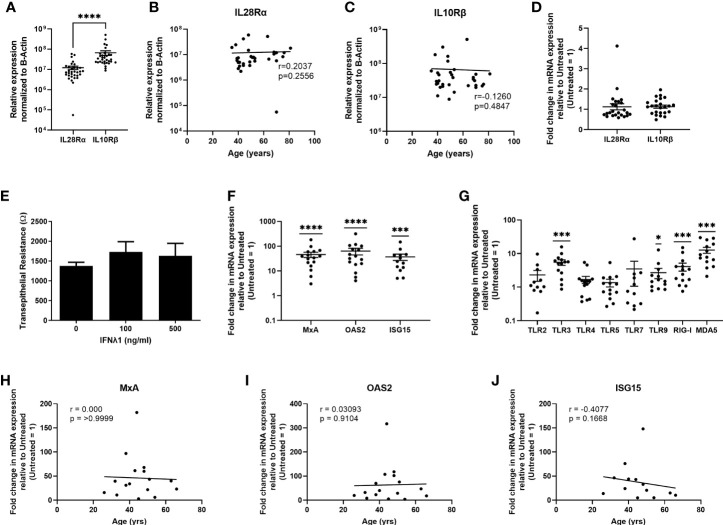
Uterine epithelial cells express Type III IFN receptors and respond to IFNλ1 by upregulating ISGs and pattern recognition receptors. **(A)** mRNA from uterine epithelial cells was recovered and analyzed for relative expression of IL28Rα and IL10Rβ normalized to β-Actin. Each circle represents an individual patient (*n = 33*). Values for IL28Rα **(B)** and IL10Rβ **(C)** from panel A were plotted against patient age. Each circle represents an individual patient (*n = 33*). **(D)** Fold change in mRNA expression of IL28Rα and IL10Rβ following stimulation of primary uterine epithelial cells with poly (I:C) (25ug/ml) for 24 hrs. Each circle represents an individual patient (*n = 24*). **(E)** Transepithelial resistance of primary uterine epithelial cells following dose-response of IFNλ1 (100, 500 ng/ml) stimulation for 24 hrs. **(F)** mRNA expression of MxA, OAS2, and ISG15 by primary uterine epithelial cells following treatment with IFNλ1 (500 ng/ml) for 24 hrs. Untreated control = 1. Each circle represents an individual patient *(n = 13-16*) **(G)** mRNA expression of TLR2, TLR3, TLR4, TLR5, TLR7, TLR9, RIG-I, and MDA5 by primary uterine epithelial cells following treatment with IFNλ1 (500 ng/ml) for 24 hrs. Untreated control = 1. Each circle represents an individual patient (*n = 14*). **(H–J)** Values for MxA, OAS2, and ISG15 mRNA expression from panel **(F)** were plotted against patient age. Each circle represents an individual patient *(n = 13-16*). mRNA expression data in panels **(A–C)** is normalized to the housekeeping gene β-Actin. mRNA expression data in panels **(D–I)** is normalized to the housekeeping gene β-Actin, and then further normalized to the untreated control whose value is then set to 1. Data is shown as mean +/- SEM. Each circle represents an individual patient. Wilcoxon matched-pairs signed rank test **(A)**. Non-parametric Spearman correlation analysis **(B, C, H–J)**. One-sample non-parametric Wilcoxon test **(F, G)** *p < 0.05; ***p < 0.001; ****p < 0.0001.

Key to IFNλ1 sensitivity is the expression of its heterodimeric receptor complex composed of IL28Rα (IFNLR1) and IL10Rβ (IL10R2). While IL28Rα is exclusively used by the Type III IFNs, IL10Rβ is shared with other members of the IL10 family (3). Therefore, we hypothesized that IL28Rα expression would be lower than IL10Rβ. Analysis of mRNA obtained from uterine epithelial cells demonstrated that they express IL28Rα and IL10Rβ, with IL10Rβ being expressed at significantly higher levels than IL28Rα (**Figure 5A**), with no effect of age on the expression of either receptor (**Figures 5B, C**), suggesting that uterine epithelial cells can respond to IFNλ1 present in the extracellular environment. To determine if poly(I:C) could upregulate the expression of either subunit in epithelial cells as part of the antiviral response, we measured the expression of IL28Rα and IL10Rβ following 24 hrs of stimulation with poly (I:C). As seen in **Figure 5D**, poly (I:C) had no effect on expression of either subunit by epithelial cells.

To determine if epithelial cells respond to IFNλ1, we stimulated ECC-1 cells for 24 hrs IFNλ1 (1-1000ng/ml) and measured ISG expression (**Supplementary Figure 1A**). Maximal upregulation of ISG expression was at 1000 ng/ml of IFNλ1 with MxA, OAS2 and ISG15 increasing by approximately 3-, 18-, and 8-fold respectively. There was also no effect of IFNλ1 on barrier function of ECC-1 cells as measured by transepithelial resistance (**Supplementary Figure 1B**). Building on our ECC-1 findings, we stimulated primary uterine epithelial cells with IFNλ1 (500ng/ml) for 24 hrs. Similar to ECC-1 cells, IFNλ1 had no effect on epithelial barrier function as measured by transepithelial resistance (**Figure 5E**). We then measured the expression of MxA, OAS2, and ISG15. As shown in **Figure 5F**, uterine epithelial cells significantly upregulated MxA, OAS2 and ISG15 by an average of approximately 46-, 63- and 37-fold respectively. We also measured the effect of IFNλ1 on the expression of pattern recognition receptors (PRRs) which are essential for the detection of incoming viral, bacterial, and fungal pathogens. IFNλ1 significantly upregulated expression of the viral PRRs TLR3, TLR9, RIG-I and MDA5 by approximately 5-, 3-, 4- and 13-fold respectively (**Figure 5G**), but had no effect on the expression of TLR2, TLR4, TLR5, and TLR7. As seen in **Figures 5H–J**, IFNλ1 had no effect on MxA, OAS2 or ISG15 expression with increasing age.

**Figure 6:**
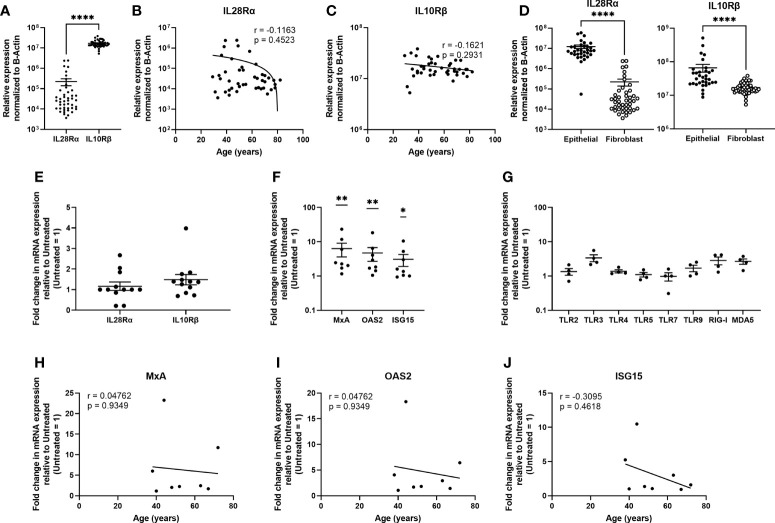
Uterine stromal fibroblasts express Type III IFN receptors and respond to IFNλ1 by upregulating ISGs and pattern recognition receptors. **(A)** mRNA from uterine stromal fibroblasts was recovered and analyzed for relative expression of IL28Rα and IL10Rβ normalized to β-Actin. Each circle represents an individual patient (*n = 44*). Values for IL28Rα **(B)** and IL10Rβ **(C)** from panel A were plotted against patient age. Each circle represents an individual patient (*n = 44*) **(D)** Comparison of IL28Rα and IL10Rβ mRNA expression between non-matched uterine epithelial cells (*n = 33*) and uterine stromal fibroblasts (*n = 44*). Each circle represents an individual patient. **(E)** Fold change in mRNA expression of IL28Rα and IL10Rβ following stimulation of uterine stromal fibroblasts with poly (I:C) (25ug/ml) for 24 hrs. Each circle represents an individual patient (*n = 12*). **(F)** mRNA expression of MxA, OAS2, and ISG15 by primary uterine stromal fibroblasts following treatment with IFNλ1 (500 ng/ml) for 24 hrs. Untreated control = 1. Each circle represents an individual patient (*n = 8*) **(G)** mRNA expression of TLR2, TLR3, TLR4, TLR5, TLR7, TLR9, RIG-I, and MDA5 by primary uterine stromal fibroblasts following treatment with IFNλ1 (500 ng/ml) for 24 hrs. Untreated control = 1. Each circle represents an individual patient (*n = 4*). **(I–K)** Values for MxA, OAS2, and ISG15 mRNA expression from panel **(F)** were plotted against patient age. Each circle represents an individual patient (*n = 8*). mRNA expression data in panels **(A–D)** is normalized to the housekeeping gene β-Actin. mRNA expression data in panels **(E, F–J)** is normalized to the housekeeping gene β-Actin, and then further normalized to the untreated control whose value is set to 1. Data is shown as mean +/- SEM. Each circle represents an individual patient. Wilcoxon matched-pairs signed rank test **(A)**. Non-parametric Spearman correlation analysis **(B, C, H–J)**. Mann-Whitney t-test **(D)**. One-sample non-parametric Wilcoxon test **(E, F, G)**. *p < 0.05; **p < 0.01; ****p < 0.0001.

Similar to the epithelial cells, uterine stromal fibroblasts expressed IL28Rα and IL10Rβ, with significantly higher mRNA levels of IL10Rβ compared to IL28Rα (**Figure 6A**) and no effect of age on their expression (**Figures 6B, C**). When compared to epithelial cells, fibroblasts expressed significantly lower levels of both subunits (**Figure 6D**). Poly (I:C) had no effect on the expression of either subunit (**Figure 6E**). Following stimulation with IFNλ1 (500ng/ml) for 24 hrs, fibroblasts significantly upregulated expression of MxA, OAS2, and ISG15. However, in comparison to epithelial cells, fibroblasts were weakly sensitive to the presence of IFNλ1 (500ng/ml), with MxA, OAS2 and ISG15 upregulation by 6.3-, 4.7-, and 3.1-fold (**Figure 6F**). Similarly, fibroblasts only weakly upregulated TLR3, TLR9, RIG-I and MDA5 (**Figure 6H**). As shown in **Figures 6H–J**, IFNλ1 had no effect on MxA, OAS2 or ISG15 upregulation in stromal fibroblasts with increasing age.

Beyond IFNλ1, poly (I:C) stimulation of uterine epithelial cells and fibroblasts also leads to increased secretion of a broad range of inflammatory cytokines and antimicrobials such as human beta defensin 2 (HBD2), elafin, RANTES, CCL4 and CCL20 (29, 32–34). In addition to being cytokines, these proteins also inhibit survival of pathogens such as HIV (19, 21, 32, 33). To investigate whether poly (I:C)-induced IFNλ1 secretion could be responsible for the upregulation of these antimicrobials and cytokines, we measured the secretion of the HBD2, RANTES, CCL20, elafin and CCL4 in response to IFNλ1 (500ng/ml for 24 hrs). There was no effect of IFNλ1 stimulation on the secretion of HBD2, elafin, RANTES, CCL20 and CCL4 by either epithelial cells or fibroblasts (**Supplementary Figure 2**). This suggests that the primary effect of IFNλ1 is to upregulate the intracellular expression of antiviral ISGs.

### Estradiol Inhibits IFNλ1 Signaling in Epithelial Cells

**Figure 7:**
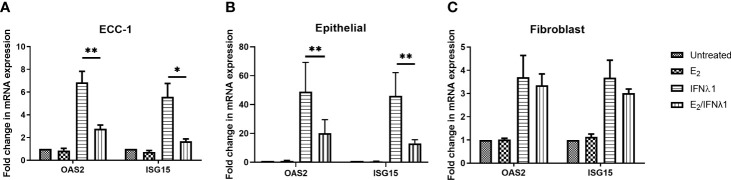
Estradiol (E_2_) inhibits IFNλ1 signaling in ECC-1 cells and uterine epithelial cells. **(A)** Polarized ECC-1 cells (*n = 5*), **(B)** uterine epithelial cells (*n = 8*) and **(C)** uterine fibroblasts (*n = 3*) were pretreated with E_2_ (5x10^-8^M) for 48 hrs after which, media was replenished in the presence or absence of IFNλ1 (500 ng/ml) for a further 24 hrs. During this period, E_2_ was maintained in the culture media. Subsequently, mRNA was analyzed for OAS2 and ISG15 expression by RT-PCR. mRNA expression data is presented as mean-fold +/- SEM change in gene expression over untreated control and is normalized to the housekeeping gene β-Actin, and then further normalized to the untreated control whose value is set to 1. Wilcoxon matched-pairs signed rank test. *p < 0.05; **p < 0.01.

Since neither E_2_ or P altered the upregulation of IFNλ1 in response to poly (I:C), we asked whether E_2_ could affect the stimulatory effect of IFNλ1 on ISG expression by epithelial cells or fibroblasts. We pretreated uterine epithelial cells, fibroblasts, and ECC-1 cells with E_2_ (5x10^-8^M) for 48 hrs prior to treatment with IFNλ1 (500ng/ml) for a subsequent 24 hrs in the presence or absence of E_2_. E_2_ significantly reduced the upregulation of OAS2 and ISG15 mRNA in ECC-1 cells after IFN treatment from approximately 6- and 4-fold to below 2-fold respectively (**Figure 7A**). A similar inhibitory effect on OAS2 and ISG15 was observed in primary uterine epithelial cells treated with under the same conditions (**Figure 7B**). In contrast, there was no E_2_ effect in uterine fibroblasts (**Figure 7C**).

**Figure 8:**
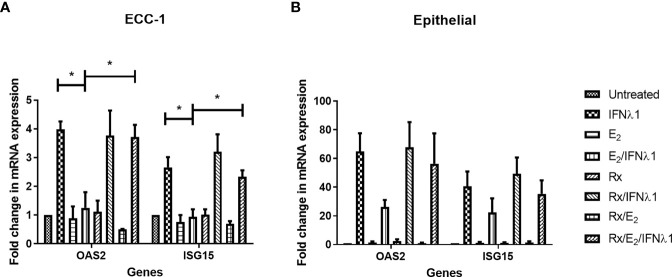
Estradiol (E_2_) inhibits IFNλ1-induced upregulation of OAS2 and ISG15 in ECC-1 cells and uterine epithelial cells *via* ERα. **(A)** Polarized ECC-1 cells (*n = 3*) or **(B)** uterine epithelial cells (*n = 3*) were pretreated with Raloxifene (Rx) (5x10^-6^M) for 1 hr prior to introduction of E_2_ (5x10^-8^M) for a subsequent 48 hrs. Following washout, Rx, E_2_ or IFNλ1 (500 ng/ml) were added to the cells in the combinations listed for a further 24 hrs prior to mRNA analysis of OAS2 and ISG15 expression by real-time RT-PCR. mRNA expression data is presented as mean-fold +/- SEM change in gene expression over untreated control and is normalized to the housekeeping gene β-Actin, and then further normalized to the untreated control whose value is set to 1. Wilcoxon matched-pairs signed rank test. *p < 0.05.

E_2_ exerts its effects *via* estrogen receptor (ER) α and β. To determine the pathway of E_2_ action in ECC-1 cells, we used the selective estrogen response modulator, Raloxifene (Rx), which blocks ERα signaling. Pretreatment of ECC-1 cells with Rx (5x10^-6^M, 48 hrs) blocked the inhibitory effects of E_2_ on the upregulation of OAS2 and ISG15 after treatment with IFNλ1 **(** **Figure 8A**). Thus, direct E_2_-ERα interactions can block IFNλ1 signaling and potentially inhibit the antiviral response.

As a part of these studies, Rx was used to block E_2_ binding to ERα in primary uterine epithelial cells. As seen in **Figure 8B**, in primary epithelial cells Rx blocked the suppression of OAS2, and ISG15 by E_2_ following treatment with IFNλ1.

### Progesterone Selectively Enhances Upregulation of ISG15 by Epithelial Cells

**Figure 9:**
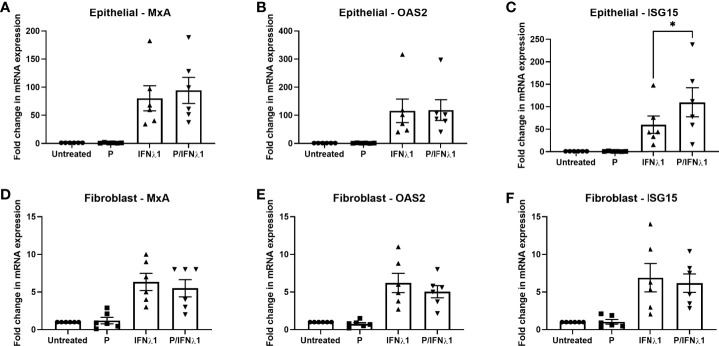
Progesterone (P) enhances IFNλ1-induced upregulation of ISG15 in uterine epithelial cells. Polarized uterine epithelial cells **(A–C)** (*n = 6*) or uterine fibroblasts **(D–F)** (*n = 6*) were pretreated with P (1x10^-7^M) for 48 hrs after which media was replenished in the presence or absence of IFNλ1 (500 ng/ml) for a further 24 hrs. During this period, P was maintained in the culture media. Subsequently, mRNA was analyzed for MxA **(A, D)**, OAS2 **(B, E)**, or ISG15 **(C, F)** expression by RT-PCR. Each symbol represents an individual patient. mRNA expression data is presented as mean-fold +/- SEM change in gene expression over untreated control and is normalized to the housekeeping gene β-Actin, and then further normalized to the untreated control whose value is set to 1. Wilcoxon matched-pairs signed rank test. *p < 0.05.

We then investigated whether P could modulate the IFNλ1-induced upregulation of MxA, OAS2, or ISG15. Epithelial cells and fibroblasts were treated with P (1x10^-7^M) for 48 hrs prior to treatment with IFNλ1 (500ng/ml) for a subsequent 24 hrs in the presence or absence of P. As seen in **Figure 9**, P had no effect on the IFNλ1-induced upregulation of MxA or OAS2 in either epithelial cells (**Figures 9A, B**) or fibroblasts (**Figures 9D, E**). However, P did significantly potentiate the upregulation of ISG15 by epithelial cells following IFNλ1 treatment (**Figure 9C**). In contrast, there was no effect of P on the IFNλ1-induced upregulation of ISG15 by fibroblasts (**Figure 9F**). Together this data shows that the effects of P are selective to specific ISGs and cell type.

## Discussion

We found that primary human uterine epithelial cells, uterine fibroblasts, and ECC-1 uterine epithelial cells rapidly upregulate IFNλ1 expression and secretion in response to the viral mimic poly (I:C), independently of E_2_ or P. The quantity of IFNλ1 secreted following poly (I:C) treatment was significantly greater by epithelial cells obtained from older women compared to younger women. In contrast, there was no effect of age on the secretion of IFNλ1 by fibroblasts. IFNλ1 in turn induced the expression of antiviral ISGs and viral PRRs in epithelial cells, but only weakly in fibroblasts. E_2_ inhibited the upregulation of MxA, OAS2 and ISG15 by IFNλ1 in epithelial cells *via* ERα but had no effect in uterine fibroblasts. Lastly, P had no effect on MxA and OAS2 expression by either epithelial cells or fibroblasts following IFNλ1 treatment. However, P did significantly increase the upregulation of ISG15 by epithelial cells following treatment with IFNλ1 compared to IFNλ1-treated cells alone.

A fundamental role of the innate immune system is the recognition and response to pathogens present in the external environment. As part of this response, the secretion of IFNs is essential for the induction and propagation of an immune response to viral pathogens *via* the upregulation of ISGs. We have shown, for the first time, that in response to poly (I:C), IFNλ1 is upregulated in primary uterine epithelial cells and stromal fibroblasts. These findings extend our understanding of the role of IFNλ1 since other studies have shown that poly (I:C) upregulates the expression of IFNλ1 in blood-derived dendritic cells, macrophages, microglia and epithelial cells (nasal, intestinal and alveolar) (13–15, 35). The upregulation of IFNλ1 in response to poly (I:C) demonstrates that IFNλ1 is a part of the early innate immune response to viral pathogens in the uterine endometrium. This builds upon our earlier studies showing that IFNβ (25) and IL-27 (26), which are also stimulators of ISG expression, are upregulated by poly (I:C) in uterine epithelial cells and fibroblasts. Together, these studies demonstrate that viral exposure in the uterine endometrium leads to an immune response characterized by the upregulation antiviral cytokines such as IFNλ1, IFNβ, and IL-27 which in turn can induce the expression of antiviral ISGs.

While the role of Type III IFNs in protecting the mucosal surfaces, particularly the gastrointestinal and lung mucosa is well established, increasing evidence demonstrates that Type III IFNs protect the FRT from incoming pathogens. Studies have shown that IFNλ1 protects the placenta from bacterial (36) and viral infections including ZIKV (17, 18). Blockade of Type III IFN signaling in an endocervical epithelial cell line leads to increased infection by HSV-2 (15). In addition, IFNλ1 and IFNλ2 also reduce HIV infection of macrophages, CD4+ T cells and peripheral blood lymphocytes (37–40). Thus, the potential exists for IFNλs to play a central role in determining the outcome of HIV transmission, as well as other STIs, that enter the FRT. While we have not addressed whether IFNλ1 secretion and signaling prevent infection by pathogens of uterine epithelial cells and fibroblasts, it is likely that the increased expression of ISGs creates an antiviral state within the cells that reduces the possibility of successful infection.

Aging is linked to altered immune function in the FRT (41) but whether increased age leads to a decrease in immune protection in the FRT is unclear. Previous studies by our group have shown that antibacterial activity of apical uterine epithelial cell secretions against *Staphylococcus aureus* decrease after menopause (42). In the current study, we show for the first time that increased age leads to an increase in IFNλ1 secretion by uterine epithelial cells, but not stromal fibroblasts, following stimulation with the viral mimic poly (I:C). The mechanism behind this increased secretion is unknown, but our observation could suggest that the contribution of IFNλ1 to antiviral protection of the uterine epithelium increases with age. Whether aging has a similar effect with other Type III IFNs requires further study. As we observed no effect of sex hormones on the secretion of IFNλ1, it is unlikely that the reduced levels of sex hormones in post-menopausal women are responsible for the increased secretion following poly (I:C) stimulation.

Since IFNλ1 is secreted apically but not basolaterally by uterine epithelial cells, our findings suggest that IFNλ1 secretion by an epithelial cell is primarily targeted towards adjacent epithelial cells lining the FRT to enhance intracellular protection following pathogen exposure. Additionally, increased IFNλ1 secretion by uterine epithelial cells may compensate for decreased immune protection elsewhere in the FRT (34, 41, 43). Further studies are required to understand the mechanistic basis for differential effects of menopause and aging on IFNλ1 secretion in the different tissues and cell types in the FRT.

In contrast to the increased secretion of IFNλ1 by epithelial cells with age, we found no effect of age on the sensitivity of both uterine epithelial cells and fibroblasts to IFNλ1 stimulation. This suggests that while the production of IFNλ1 by epithelial cells changes with age, the ability of IFNλ1 to induce an antiviral response does not, particularly since the expression of the Type III IFN receptors, IL28Rα and IL10Rβ, also do not change with age. The lack of an aging effect on IFNλ1-induced ISG expression in uterine epithelial cells and fibroblasts does not preclude age-dependent effects on IFNλ1 signaling in other cell types in the FRT, at other mucosal sites, or by other Type III IFNs. However, there are several important caveats to consider in our study. First, is our small population size, particularly for the IFNλ1 stimulation experiments, where we used epithelial cells and fibroblasts from 16 and 8 different women respectively. Given this small population size, and the wide range in ISG expression in our results, any aging effects may not be detectable unless a larger population cohort is used. Second, our analyses were performed after 24 hrs of IFNλ1 stimulation and more extensive time course studies may be useful to determine whether aging affects the response to IFNλ1 beyond 24 hrs, particularly since some studies suggest that Type III IFNs are important in maintaining the antiviral response over longer time periods (44, 45). Third, we used a high dose of IFNλ1 which may have masked any aging effects. Whether aging could exert an effect on gene expression in the presence of a lower concentration of IFNλ1 is important to define. Lastly, we measured ISG mRNA, but not protein, expression in response to IFNλ1 stimulation. While unlikely, it is possible that the mRNA expression does not correlate with protein levels. These questions should be addressed in future studies.

The premenopausal and pregnant uterine endometrium is exposed to changing concentrations of sex hormones that regulate multiple aspects of innate and adaptive immunity within the FRT, including cytokine and antimicrobial secretion, barrier function and immune cell numbers (1). While the secretion of IFNλ1 was unaffected by E_2_ or P, both hormones altered the actions of IFNλ1 on ISG upregulation in uterine epithelial cells. In response to IFNλ1, E_2_ suppresses the upregulation of OAS2, and ISG15 by uterine epithelial cells, while P enhances the expression of ISG15. That this stimulatory effect of P was not observed with MxA or OAS2 emphasizes the unique regulatory control of intracellular innate protection in epithelial cells by sex hormones. Others have shown that ISG15 is upregulated at the time of implantation (46, 47) which suggests that it may function in a way that transcends its antiviral effects (48). Our finding builds on this study by demonstrating that P can further increase ISG15 expression following IFNλ1 stimulation through direct effects on epithelial cells in the uterine endometrium. To the best of our knowledge, these findings are the first demonstration that the actions of IFNλ1 in the human uterus are regulated by sex hormones. This suggests that the contribution of IFNλ1 to innate immune protection may vary across the menstrual cycle and at different stages of pregnancy.

We have previously hypothesized that immune protection against HIV and other sexually transmitted infections is downregulated in the uterus at mid-secretory phase of the menstrual cycle (49). Several studies have shown that transmission of pathogens in the FRT varies with sex hormone levels and menstrual cycle stage. For example, vaginal infection of pigtail macaques by SHIV is higher during secretory phase than proliferative phase (50–52). Similarly, HIV infection is greater in cervico-vaginal explants recovered from secretory phase (51). In ovariectomized mice and *in vitro* there is increased vaginal HSV-2 transmission following treatment with progesterone (53, 54) while in other studies using ovariectomized mice treated with IFNλ1, vaginal, cervical, and uterine ZIKV infection was greater in mice treated with P alone, compared to mice treated with both E_2_ and P (55). Our findings suggest that the modulatory effect of sex hormones on the action of IFNλ1 can potentially decrease protection against incoming pathogens, and thus increase the risk of successful transmission of pathogens that enter the FRT. However, whether the IFNλ1-mediated antiviral response by uterine epithelial cells is capable of protecting the uterine endometrium against incoming pathogens, and whether its potential contribution changes across the menstrual cycle, is unknown and not addressed in our study. Future studies will need to address whether exposure to IFNλ1 prevents the infection of uterine epithelial cells and fibroblasts by relevant pathogens.

Beyond the FRT, whether sex hormones modulate IFNλ1-mediated responses at mucosal sites outside the FRT such as the lung, skin, and gastrointestinal tracts and thus alter protection against pathogens that enter at these surfaces remains to be determined but is of considerable importance given the ubiquity of pathogens that can enter the body at these locations. Recognizing that the FRT is exposed to higher levels of sex hormones than other mucosal surfaces (56–61), future studies will need to use lower doses of sex hormones that are representative of levels seen at these sites.

Of particular interest is whether, in addition to being upregulated as part of the innate immune response, the secretion or effects of IFNλ1 are increased at the time of implantation when the semi-allogeneic fertilized egg contacts uterine epithelial cells, fibroblasts and decidual cells at the implantation site. Basic reproductive processes are often accompanied by a limited degree of inflammation and Type I IFNs, such as IFNτ, are thought to be important in mediating uterine receptivity in mammals (62). In animal models, Type I IFNs such as IFNτ are upregulated to as part of the implantation process (63). In turn, IFNs induce ISGs such as ISG15 which are essential for successful implantation (46, 47). Since implantation occurs when P levels are high, this could explain why P enhanced the expression of ISG15 following stimulation of epithelial cells with IFNλ1. Whether Type III IFNs have a similar function in regulating the expression of a larger panel of ISGs as part of the reproductive process is unclear.

As part of the reproductive process, the immune system in the FRT is precisely regulated to achieve a balance between immune tolerance which allows the survival of allogeneic sperm and potentially a semi-allogeneic fetus, while maintaining immune protection against external pathogens. High levels of E_2_ as seen during ovulation and mid-secretory phase in the non-pregnant endometrium could reduce the innate antiviral response increasing the chances for successful fertilization. The dose of P (1x10^-7^M) at which we observed a stimulatory effect on IFNλ1-induced ISG15 expression is representative of levels at mid-secretory phase in the endometrium when implantation occurs. Therefore, P may modulate IFNλ1-induced ISG15 expression as part of a successful implantation program.

Through its intracellular receptors, E_2_ is a potent modulator of immune function in the FRT (1). Our investigation of the effects of E_2_ on IFNλ1-induced gene expression demonstrate reduced the upregulation of OAS2 and ISG15 by IFNλ1 *via* ERα. While the precise regulation of IFNλ1 signaling downstream of ERα remains unclear, binding of IFNλ1 to IL28Rα and IL10Rβ leads to the phosphorylation of JAK2 as part of the signal transduction cascade (64, 65). In HuH7 and T-47D cells, E_2_ inhibits growth hormone-induced signaling by preventing phosphorylation of JAK2 *via* upregulation of Suppressor of Cytokine Signaling (SOCS) 2 (66). Therefore, disruption of JAK-mediated signaling may be a possible mechanism for E_2_-mediated inhibition of IFNλ1 in uterine epithelial cells.

Consistent with our results previous studies have shown that IFNλ1 expression is regulated by Type I IFNs, and is therefore an ISG itself (31). This suggests that a Type I IFN response is necessary for optimal antiviral protection in uterine epithelial cells. Other studies have shown that ISG expression induced by Type I IFNs peaks earlier and higher than ISG expression induced by Type III IFNs which maintains the antiviral response for longer (44, 45). This suggests that Type I IFNs are responsible for the upregulation of ISGs early in the antiviral response, while Type III IFNs are responsible for increased duration of the antiviral response. Since our studies were performed over an acute timepoint (24 hrs), the possibility remains that IFNλ1 is necessary for long term upregulation of ISGs but is not necessary for the immediate upregulation of ISGs following poly (I:C) stimulation of uterine epithelial cells and fibroblasts. Future studies will need to address the effects of long-term IFNλ1 stimulation on ISG expression in the FRT.

Together our results demonstrate that the FRT is a unique mucosal site in which the endocrine and immune systems intersect to create a unique and complex immune environment that balances protection with procreation. IFNλ1 is an important component of innate immune protection within the human FRT against potential viral pathogens. The selective effects of E_2_ and P on IFNλ1 function suggests that the antiviral efficacy of IFNλ may vary in women with stage of the menstrual cycle. Since IFNλs are increasingly used in a therapeutic setting, it is important to understand how endogenous (E_2_ and P) and exogenous (contraceptives and hormone therapy) hormone exposure may alter IFNλ-mediated protection both in the FRT, and at other sites in the body in women of all ages.

## Data Availability Statement

The raw data supporting the conclusions of this article will be made available by the authors, without undue reservation.

## Ethics Statement

The studies involving human participants were reviewed and approved by Committee for the Protection of Human Subjects Dartmouth-Hitchcock Medical Center. The patients/participants provided their written informed consent to participate in this study.

## Author Contributions

MVP, DCH, and FDB performed the experiments. MVP prepared the figures. MVP and CRW wrote the manuscript. All authors contributed to the article and approved the submitted version.

## Funding

Supported by NIH AI102838, AI071761, AI117739 and AG064794 (CRW).

## Conflict of Interest

The authors declare that the research was conducted in the absence of any commercial or financial relationships that could be construed as a potential conflict of interest.

## Publisher’s Note

All claims expressed in this article are solely those of the authors and do not necessarily represent those of their affiliated organizations, or those of the publisher, the editors and the reviewers. Any product that may be evaluated in this article, or claim that may be made by its manufacturer, is not guaranteed or endorsed by the publisher.

## Supplementary Material

Supplementary Figure 1 IFNλ1 upregulates MxA, OAS2, and ISG15 expression by ECC-1 cells but does not affect TER. ECC-1 uterine epithelial cells were treated recombinant human IFNλ1 (10, 100, 1000 ng/ml) for 24 hrs prior to analysis of gene expression by real-time RT-PCR **(A)** or transepithelial resistance **(B)**. Data is shown as mean +/- SEM.

Supplementary Figure 2 IFNλ1 has no effect on the secretion of HBD2, elafin, CCL20, RANTES, and CCL4 by uterine epithelial cells. Primary human uterine epithelial cells (*n = 6*) and fibroblasts (*n = 5*) were treated with recombinant IFNλ1 (500 ng/ml) for 24 hrs after which secretions were recovered and analyzed by ELISA. Data is shown as mean +/- SEM.
