# Supplementary figures and images for: Sex Hormones and Aging Modulate Interferon Lambda 1 Production and Signaling by Human Uterine Epithelial Cells and Fibroblasts
Source: Front Immunol. 2021 Sep 24;12:718380. doi: 10.3389/fimmu.2021.718380 (PMC8497887; doi:10.3389/fimmu.2021.718380)

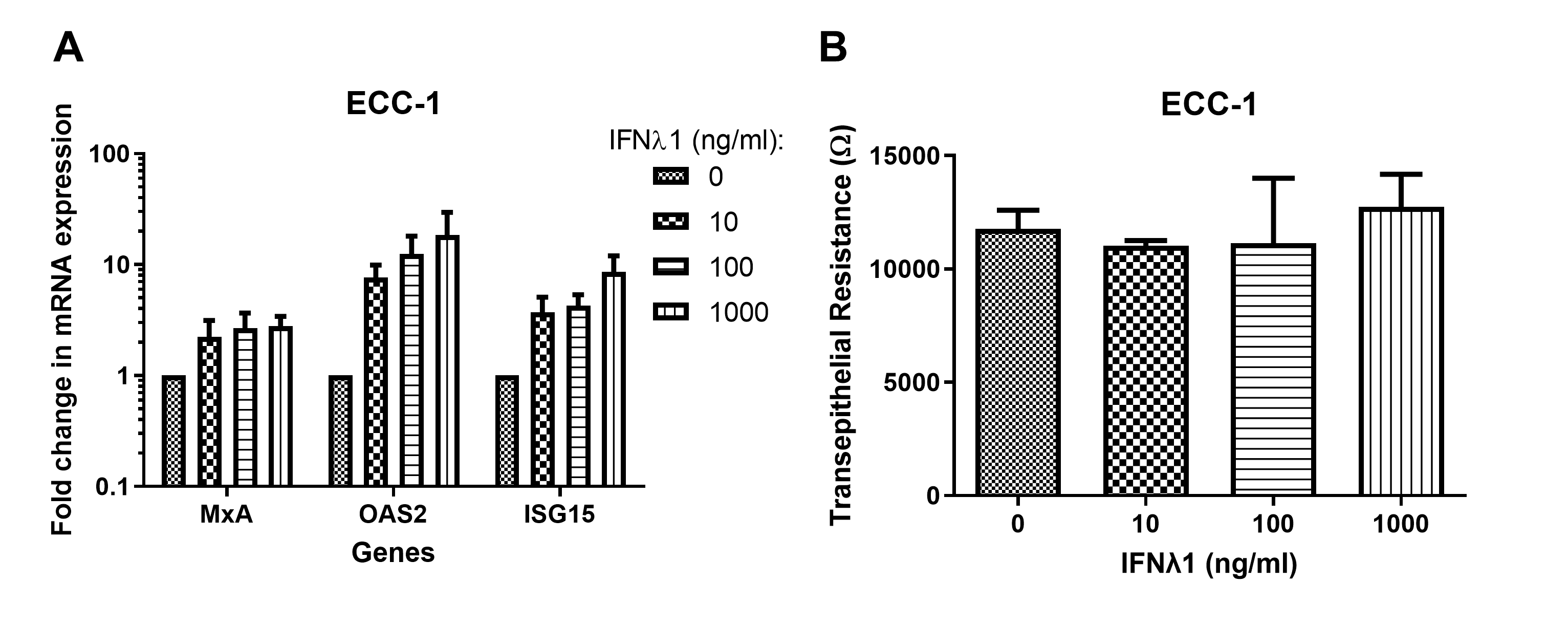

Supplement: Supplementary Figure 1 — IFNλ1 upregulates MxA, OAS2, and ISG15 expression by ECC-1 cells but does not affect TER. ECC-1 uterine epithelial cells were treated recombinant human IFNλ1 (10, 100, 1000 ng/ml) for 24 hrs prior to analysis of gene expression by real-time RT-PCR (A) or transepithelial resistance (B). Data is shown as mean +/- SEM. [file Image_1.tif]

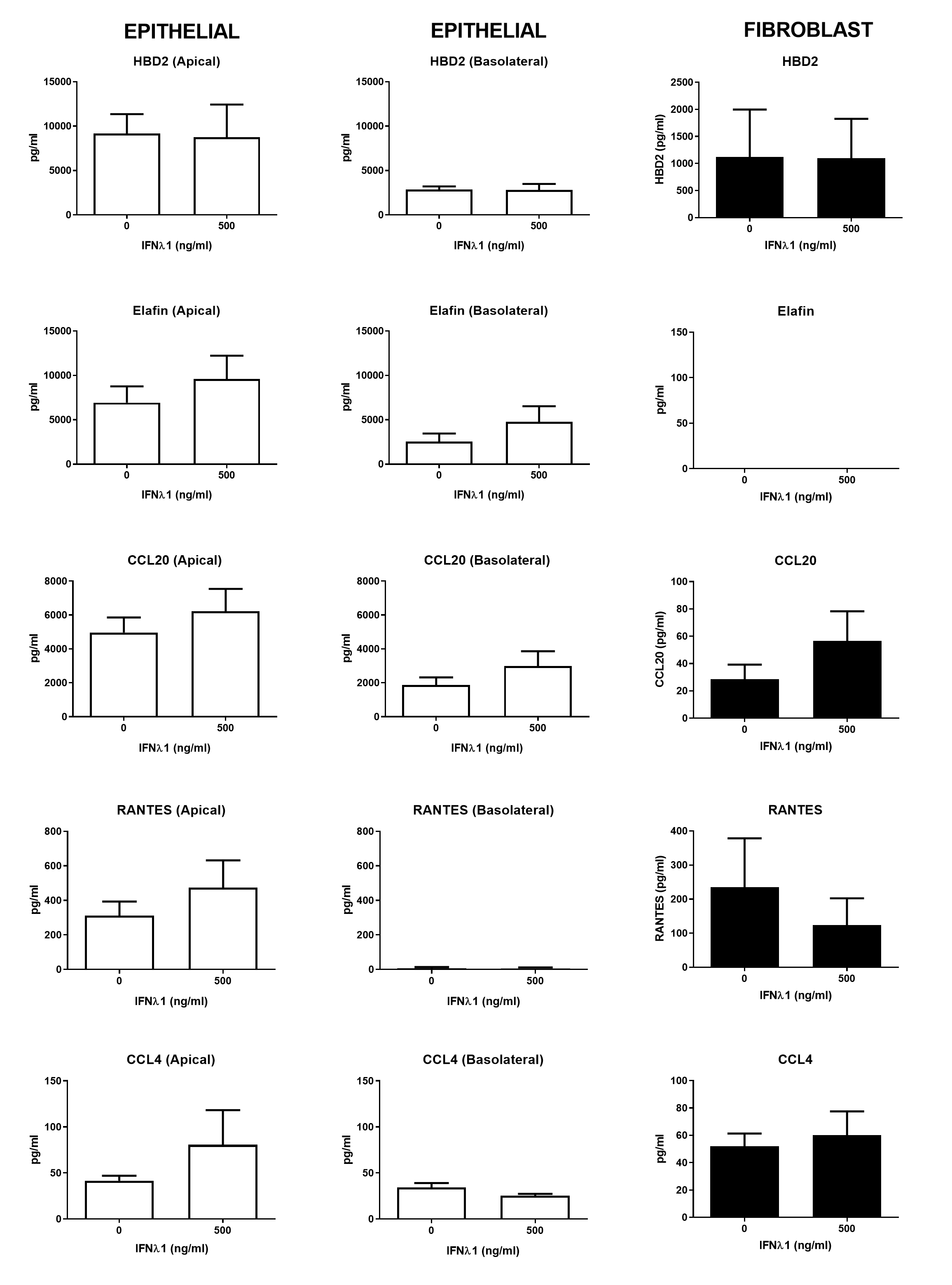

Supplement: Supplementary Figure 2 — IFNλ1 has no effect on the secretion of HBD2, elafin, CCL20, RANTES, and CCL4 by uterine epithelial cells. Primary human uterine epithelial cells (n = 6) and fibroblasts (n = 5) were treated with recombinant IFNλ1 (500 ng/ml) for 24 hrs after which secretions were recovered and analyzed by ELISA. Data is shown as mean +/- SEM. [file Image_2.tif]
